# Beware the intruder: gasodermin A as molecular guardian preventing systemic dissemination of group A streptococci following local skin infection

**DOI:** 10.1038/s41423-022-00863-4

**Published:** 2022-04-12

**Authors:** Mahyar Aghapour, Surender Surender, Dunja Bruder

**Affiliations:** 1 Infection Immunology Group, Institute of Medical Microbiology, Infection Control and Prevention, Health Campus Immunology, Infectiology and Inflammation, Otto-von-Guericke University, Magdeburg, Germany; 2 Immune Regulation Group, Helmholtz Centre for Infection Research, Braunschweig, Germany

**Keywords:** Antimicrobial responses, Innate immunity

Pyroptosis represents a host-protective mechanism that promotes clearance of pathogens by initiating the recruitment of immune cells to the site of infection. In a study recently published in *Nature*, Deng et al. extensively outlined a hitherto unknown role of the group A *Streptococcus* (GAS)-derived cysteine protease streptococcal pyrogenic exotoxin B (SpeB) in gasodermin A (GSDMA) cleavage as the primary step in induction of pyroptosis. As clearly shown in GSDMA-deficient mice infected with SpeB-producing GAS, this cleavage event is key to preventing the systemic spread and fatal course of GAS following local skin infection [1].

**Fig. 1:**
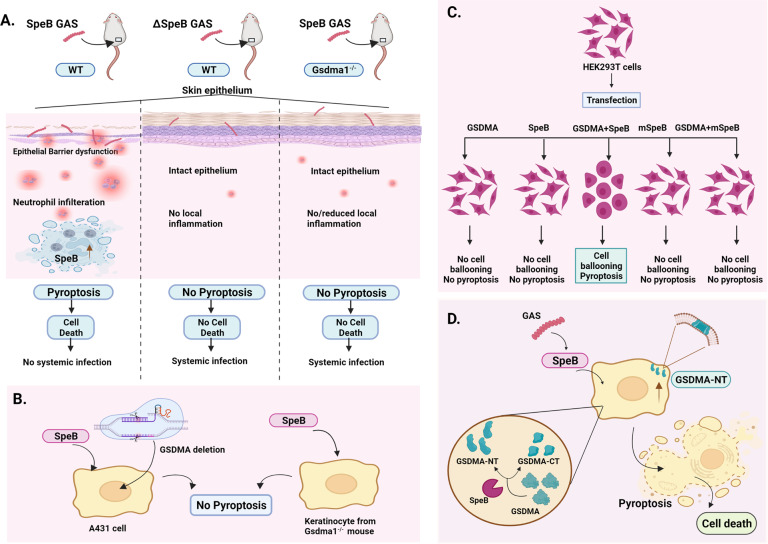
SpeB-mediated gasodermin A cleavage triggers pyroptosis in skin epithelial cells and prevents systemic dissemination of group A streptococci in mice. **A** Skin infection of wild-type mice with SpeB^+^ GAS induces local skin inflammation, and bacteria are contained at the site of infection. No or significantly reduced skin inflammation was observed following infection of wild-type mice with ΔSpeB GAS or infection of Gsdma1^−/−^ mice with SpeB^+^ GAS. Lack of skin inflammation results in impaired immune surveillance and invasive streptococcal disease. **B** GSDMA-deficient keratinocytes transfected with SpeB fail to undergo pyroptosis. **C** Cotransfection experiments in human HEK293T cells revealed that only the concerted action of functional SpeB and GSDMA is sufficient for induction of pyroptosis. **D** While GSDMA does not exhibit lipid binding and oligomerization, the N-terminal GSDMA fragment released upon SpeB cleavage oligomerizes, and due to its enhanced affinity to various lipids, it forms pores in lipid membranes. Pore formation results in cell death by pyroptosis. (Created with BioRender.com)

Multiple host-derived defense molecules regulate the induction of pyroptosis upon bacterial infection, of which the activation of caspases plays a dominant role [2]. Recognition of pathogens by innate immune sensors results in inflammasome activation, subsequently leading to caspase-dependent cleavage of gasodermins. Gasodormins comprise a family of pore-forming proteins [3]. Of these, the N-terminal GSDMD fragment was previously shown to be released following cleavage by caspases, resulting in pyroptosis [4]. Similar to GSDMD, GSDMA is expressed at high levels in keratinocytes; however, its potential contribution to pyroptosis and antibacterial immunity during GAS skin infection remains unclear and is the subject of the study highlighted here (Fig. 1).

Using a mouse infection model, Deng and colleagues revealed that in stark contrast to that of SpeB-sufficient (SpeB^+^) GAS, infection of mice with GAS lacking SpeB (ΔSpeB) failed to induce local skin inflammation. Strikingly, ΔSpeB GAS established systemic infection with considerable mortality, i.e., the presumed benefit of ameliorated local skin inflammation was eliminated by the onset of a far more devastating invasive streptococcal disease. In-depth mechanistic studies in mouse keratinocytes revealed that SpeB^+^, but not ΔSpeB, GAS induces substantial pyroptosis, directly proving a crucial role of SpeB in pyroptosis. Given its proteolytic activity, SpeB was already demonstrated to cleave host protective immunological barriers, including immunoglobulins and cell barrier junctions. Therefore, SpeB not only promotes local dissemination of GAS in mucosal surfaces but also stimulates host protective immunity.

Further mechanistic studies revealed that substantial pyroptosis occurs in SpeB^+^ GAS-infected human epidermal cells, while targeted deletion of GSDMA prevented pyroptosis, identifying GSDMA as a target of SpeB and linking it to pyroptosis. Many bacterial-derived virulence factors were reported to induce pyroptosis in a process dependent on caspase-1-mediated cleavage of GSDMD and formation of pores in cell membranes [2]. Importantly, using a cotransfection setting in combination with protease inhibitors, the authors revealed that activation of pyroptosis requires catalytically active SpeB but is independent of caspase activity. Moreover, GSDMA cleavage is exclusively activated in the presence of SpeB and not by any other GAS-derived protease, highlighting the unique specificity of GAS SpeB for GSDMA. To unequivocally demonstrate that catalytic cleavage of GSDMA by SpeB is the molecular switch activating pyroptosis, the authors again used cotransfection experiment showing that only wild-type SpeB transfected in GSDMA-expressing cells induces pyroptosis; this process was abrogated in cells expressing a mutated form of GSDMA conferring resistance to SpeB cleavage.

To further elucidate the underlying mechanism of SpeB-GSDMA-induced pyroptosis, the authors performed transfection experiments using truncated GDSMA containing either the C- or N-terminal GDSMA region. Using this approach, they identified the N-terminal GSDMA (GSDMA-NT) region to be crucial for induction of pyroptosis. To determine how SpeB-activated GSDMA-NT triggers pyroptosis, Deng and coworkers performed protein-lipid overlay assays to determine the effects of GSDMA-NT on the lipid constituents of cell membranes. Strikingly, while full-length GSDMA did not show any relevant affinity to lipids and retained its monomeric character, GSDMA-NT exhibited a strong lipid-binding capacity and formed oligomers. Consequently, enhanced lipid affinity and GSDMA-NT oligomerization led to pore formation in lipid membranes, identifying the molecular mechanism underlying SpeB-induced pyroptosis upon GAS skin infection.

To translate the in vitro data to an in vivo setting, Deng and colleagues performed additional GAS infections in mice. Since the mouse genome encodes three GSDMA homologs, the authors first confirmed that Gsdma1, which is highly expressed in mucosa and skin, is the substrate for SpeB and is involved in induction of pyroptosis. As in the human system, SpeB-cleaved Gdsma1 disrupts lipid membranes in murine keratinocytes. To ultimately verify the role of Gsdma1 in the local containment of GAS at the site of infection, Deng and colleagues performed GAS skin infections in mice lacking the *Gsdma1* gene. Consistent with their extensive in vitro data, Gsdma1-deficient mice indeed failed to develop fulminant local inflammation upon skin infection with SpeB^+^ GAS. The inability to rapidly respond to the infectious agent, however, had adverse effects on the overall course of the infection. In the absence of SpeB-induced Gsdma1 cleavage as an initiator of pyroptosis and local skin inflammation, ineffective immune containment of bacteria results in bacteremia with bacterial outgrowth in peripheral organs and substantial lethality. Of note, this phenotype can be mirrored by infection of wild-type mice with ΔSpeB GAS, again highlighting the crucial collaboration of host-derived Gasdm1 and pathogen-derived SpeB in this process.

In conclusion, Deng et al. uncovered a novel role for the GAS cysteine protease SpeB as a molecular driver of GAS-induced pyroptosis. In a one-molecule mechanism, GSDMA acts as both an innate sensor/substrate of GAS SpeB and an innate effector molecule inducing pyroptosis as an initiator of local pathogen-specific immunity. While fulminant inflammation is generally considered negative, data collected by Deng and colleagues clearly highlight its obvious benefits. Prevention of local skin inflammation due to a dysfunctional SpeB-GSDMA axis is beneficial for the host. The presumably positive effect is offset by failures in immune surveillance and containment of bacteria at the site of infection, laying the groundwork for life-threatening invasive streptococcal infection. With this in mind, one should be particularly cautious with immunosuppressive interventions in certain inflammatory settings, especially in the treatment of infection-induced inflammation. Of note, since GSDMA undergoes post-translational modification and is prone to genetic modifications [5], both of which may result in attenuation of cleaved GSDMA-NT activity and reduced pyroptosis, it is tempting to speculate that individuals with GSDMA mutations might be more susceptible to invasive GAS infection. Future studies are needed to prove or disprove this hypothesis.
